# First report of *Stemphylium lycopersici* keratitis, a complex corneal infection case

**DOI:** 10.1186/s12348-025-00505-6

**Published:** 2025-05-27

**Authors:** Zixiang Zhao, Siyu Leng, Yuhao Zou, Lijuan Xiang, Yunke Li, Yi Liu, Chongyang Wang, Man Yu

**Affiliations:** 1 Department of Ophthalmology, Sichuan Provincial People’s Hospital, University of Electronic Science and Technology of China, Chengdu, China; 2 Eye School of Chengdu, University of TCM, Chengdu, China; 3 Department of Ophthalmology, Chengdu BOE Hospital, Chengdu, China; 4 Department of Ophthalmology, Deyang People’s Hospital, Deyang, China; 5 Department of Ophthalmology, The First People’s Hospital of Yibin, Yibin, China

**Keywords:** Fungal keratitis, *Stemphylium lycopersici*, Mixed infection, New pathogenic species, Antibiotic resistance

## Abstract

Filamentous fungi are among the emerging causes of infections, although corneal infections caused by these fungi are rare, they can lead to severe clinical outcomes. In this report, we present the first documented case of keratitis caused by *Stemphylium lycopersici*, a filamentous hemipteran fungus of the Pleosporaceae family. A 66-year-old man presented conjunctival redness, irritation, and visual deterioration in his left eye, following a stone chip injury that occurred five months earlier. Despite multiple treatments, the causative pathogen remained unidentified, leading to worsening symptoms and significant vision loss. This deterioration led the patient to seek care at our hospital. An in vivo confocal microscopy (IVCM) examination suggested a fungal infection. Consequently, antifungal medications were administered, but the condition did not improve. Metagenomic next-generation sequencing (mNGS) examination of corneal scrapings revealed a mixed infection with *S. lycopersici* and human alphaherpesvirus 1. This definitive diagnosis facilitated the implementation of targeted therapy, leading to progressive symptomatic improvement. Early and rapid pathogen identification using mNGS analysis of corneal scrapings enables accurate management of infectious keratitis, contributing to visual recovery and reducing the risk of resistance to corneal pathogenic microbes.

## Background

Corneal opacities are the fifth leading cause of blindness worldwide, accounting for approximately 3.2% of all cases [1]. It is estimated that 1.5 to 2 million cases of monocular blindness are caused by corneal opacities each year, a figure likely much higher due to under-reporting in less developed countries [2]. Vision loss due to corneal opacities can be due to various factors that cause significant damage to the cornea, such as infections, inflammation, trauma, degenerative diseases, and corneal dystrophy. Among these causes, corneal infections are the most prevalent [3]. Bacteria and fungi are the most common microorganisms responsible for infectious keratitis (IK). Furthermore, mixed polymicrobial infections constitute approximately 2–15% of all cases of IK [4–6]. The diagnosis of mixed infectious keratitis can be challenging because its clinical presentation is similar to other infections [7]. The nonspecific clinical presentation, rapid progression, and pathogen-driven competition for host resources in mixed species infections pose a therapeutic challenge for clinicians. The presence of a multispecies infection can significantly complicate the progression and treatment of infectious keratitis [8].

The prevalence of fungal infections has increased in recent years due to the irrational use of antibiotics and the increasing number of patients undergoing hormonal and immunosuppressive treatments [9]. Fungal keratitis is a severe condition that often resists treatment and has a worse prognosis than bacterial keratitis [10]. Therefore, an early and accurate diagnosis of fungal keratitis is crucial to determine the appropriate treatment and achieve a cure [11]. However, since fungal cultures take longer to develop than bacterial cultures and have a low positivity rate, the early diagnosis of fungal keratitis is often delayed. Common diagnostic methods, such as sampling microscopy, fungal staining, and in vivo confocal microscopy (IVCM), have low sensitivity for early detection, thus often delaying diagnosis [12]. Clinicians can improve early diagnosis and identify the causative microorganisms by performing metagenomic next-generation sequencing (mNGS) tests on clinical specimens [13]. This approach facilitates timely and targeted treatment, aiding vision recovery, and reducing the incidence of antibiotic resistance (AMR) corneal pathogens caused by empirical treatment.

In this case report, we describe the first documented case of fungal keratitis caused by *S. lycopersici*, which was also accompanied by a mixed infection definitively linked to *human alphaherpesvirus 1*. The importance of mNGS in identifying pathogens responsible for IK, detecting dominant alterations in pathogens, and guiding the development of clinical treatment regimens is elucidated.

## Case presentation

A 66-year-old man with conjunctival redness, irritation, and visual deterioration in his left eye came to see us at our ophthalmology clinic. The patient had no history of immunological problems or diabetes. Five months ago, a stone chip entered his left eye, causing redness and a sense of a foreign body. The patient was diagnosed with a foreign body in the left eye at a local hospital. Subsequently, he underwent a foreign body debridement and received treatment with levofloxacin ophthalmic drops. After 20 days, the patient’s ocular symptoms worsened and he was treated with 0.5% levofloxacin eye drops, 0.3% tobramycin eye drops, and ganciclovir ophthalmic gel. However, his symptoms did not improve significantly. Three months later, due to the exacerbation of his symptoms and a severe decline in visual acuity, the patient visited the outpatient clinic of our hospital.

**Fig. 1:**
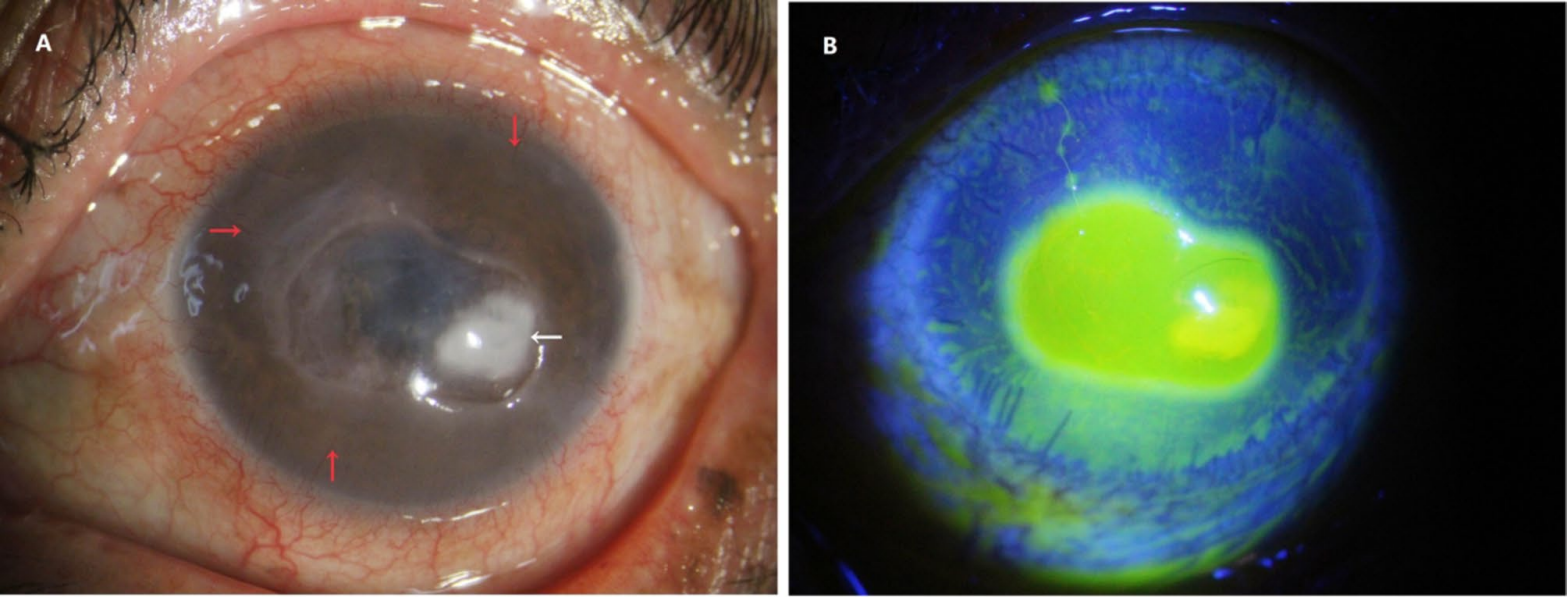
Photographs of the anterior segments during the initial visit. A substantial white abscess in the central corneal ulcer is remarkable (white arrow) and neovascularisation in the corneal limbus (red arrow). **A**, **B** Slit lamp and sodium fluorescein staining at the first visit, respectively

**Fig. 2:**
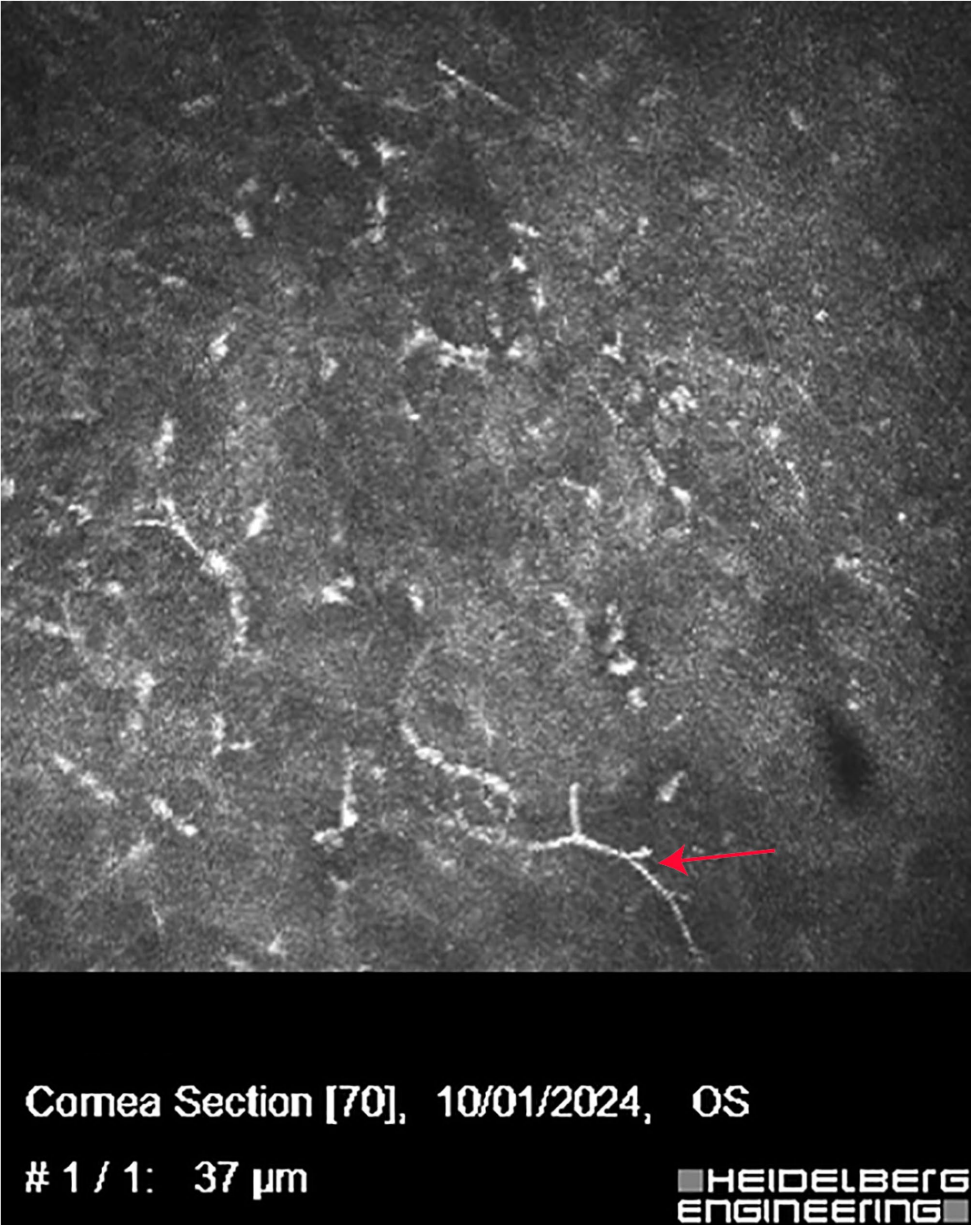
IVCM showed hyperlinear structures in the stromal layer, which implies fungal infection

On initial examination, the best corrected visual acuity in the left eye was limited to the ability to view hand movements/40 cm. A slit lamp examination showed conjunctival congestion, multiple corneal limbal neovascularization, a central map-shaped corneal ulcer measuring 4 mm ×5 mm, and a 2 mm × 3 mm white fibrinous plaque slightly off-temporal to the center, accompanied by diffuse edema of the superficial stroma layer (Fig. 1A, B). The IVCM image showed hyperlinear structures in the stroma that were typical signs of fungal hyphae, as shown in Fig. 2. The patient was diagnosed with fungal keratitis and treated with 5% natamycin eye drops every hour, 0.3% tobramycin eye drops three times daily, and ganciclovir ophthalmic gel three times daily. Despite intensive topical treatments, the ulcer continued to expand. Due to the progression of the disease and the persistent lack of evidence of a particular pathogen, corneal scraping, and mNGS were taken into account for an additional study.

### Clinical course and microbiological studies

A sample of the excised host cornea was sent to the Sichuan Provincial People’s Hospital for fungal and bacterial culture, and another sample of no less than 1 mm^3^ in volume was sent to Beijing Zhide Medical Laboratory for pathogen detection using mNGS. The total genomic DNA from the scrapings was extracted using the QIAamp DNA Micro Kit (QIAGEN, Hilden, Germany, Cat# 56304) and purified. DNA libraries were prepared with the QIAseq™ Ultralow Input Library Kit (QIAGEN, Hilden, Germany, Cat# 180492). Concentration and quality were evaluated using Qubit 4.0 (Thermo Fisher Scientific, Massachusetts, USA, Cat# Q33238, RRID: SCR_018095) and Qsep-1 (Bioptic Inc., Taiwan, China). The qualified libraries were pooled and sequenced on the MGISEQ-200 platform (50 bp single-ended reads) (MGI, Shenzhen, China). After filtering for quality, low-complexity reads and human DNA, the remaining reads were aligned to the microbial genome database (ftp://ftp.ncbi.nlm.nih.gov/gen-omes/) using the Burrows-Wheeler alignment. In parallel with samples per batch, negative and positive controls were also set for mNGS detection with the same procedure and bioinformatics analysis. The specific reads number and reads per million (RPM) of each detected pathogen were respectively calculated. A positive mNGS result was defined when the microorganism was not detected in the negative control (“No template” control, NTC) and genome coverage of detected sequences belonged to this microorganism ranked top10 of the same kind of microbes or when its ratio of RPMsample to RPMNTC was (RPMsample/RPMNTC) > 10 if the RPMNTC ≠ 0. mNGS analysis revealed a mixed infection with predominant sequences attributed to S. lycopersici and human alphaherpesvirus 1(GenBank accession numbers GCA-003268315.1 and GCA-000859985.2 respectively). Specifically, S. lycopersici with a relative abundance of 94.91%, and human alphaherpesvirus 1 with a relative abundance of 95.45%. The culture results were negative, indicating nonculturable pathogens.

**Fig. 3:**
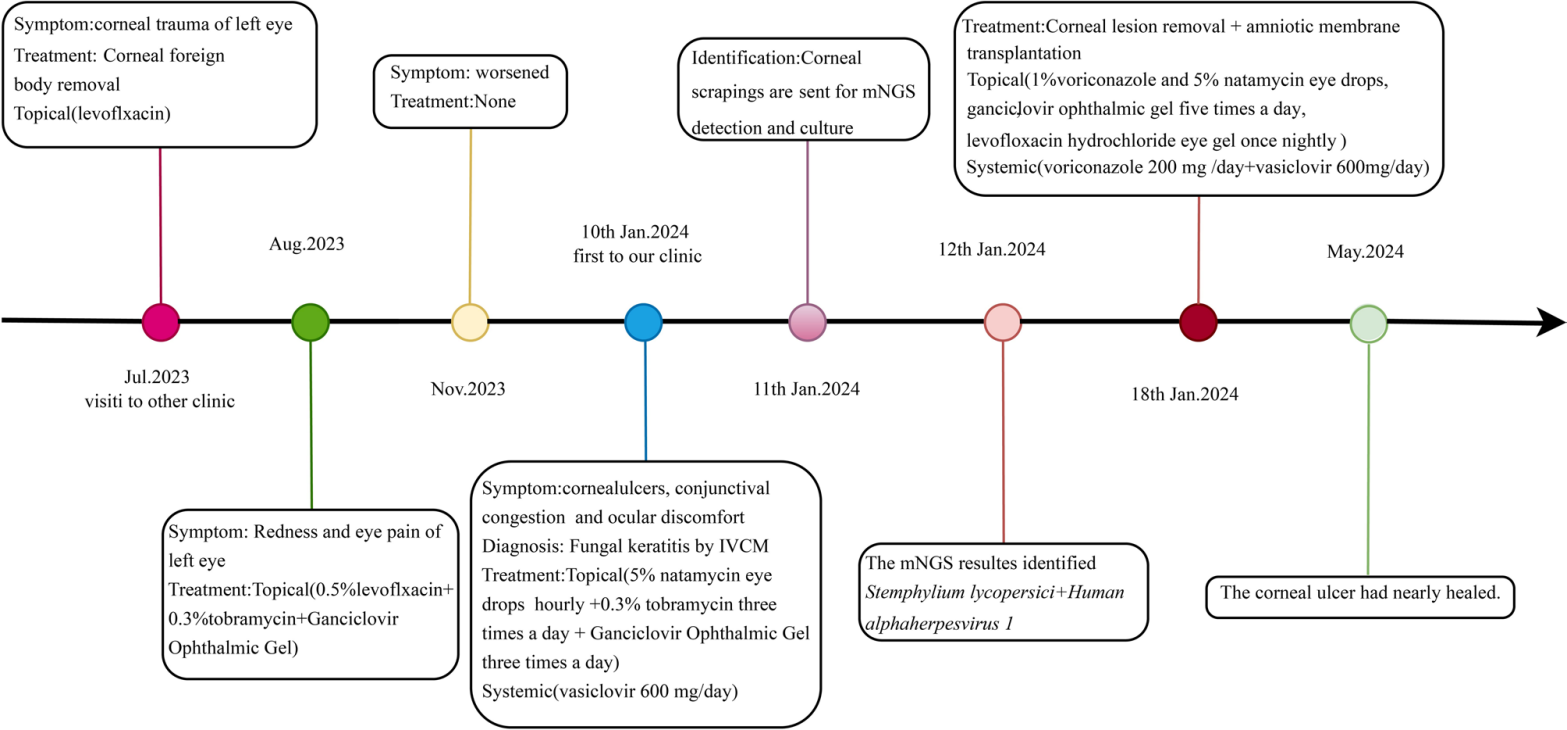
Timeline of the patient’s disease progression and the corresponding treatment

Previous studies and in vitro experiments have shown that *S. lycopersici* exhibits a favorable response to triazole antifungal agents and natamycin [14–16], which is consistent with the therapeutic results observed in our patient. The treatment regimen included the topical administration of 1% voriconazole and 5% natamycin eye drops hourly, ganciclovir ophthalmic gel five times a day, levofloxacin hydrochloride eye gel once nightly, intravenous voriconazole 200 mg twice daily, and oral aciclovir 300 mg twice a day. Subsequently, 0.1% voriconazole was injected into the matrix surrounding the corneal ulcer and the conjunctiva, respectively. Due to the extensive nature of the lesions, the patient underwent a procedure to remove the left corneal lesion and received an amniotic membrane transplantation [17]. After three months, the corneal ulcer had almost healed. The timeline of his disease progression is shown in Fig. 3.

## Discussion

**Fig. 4:**
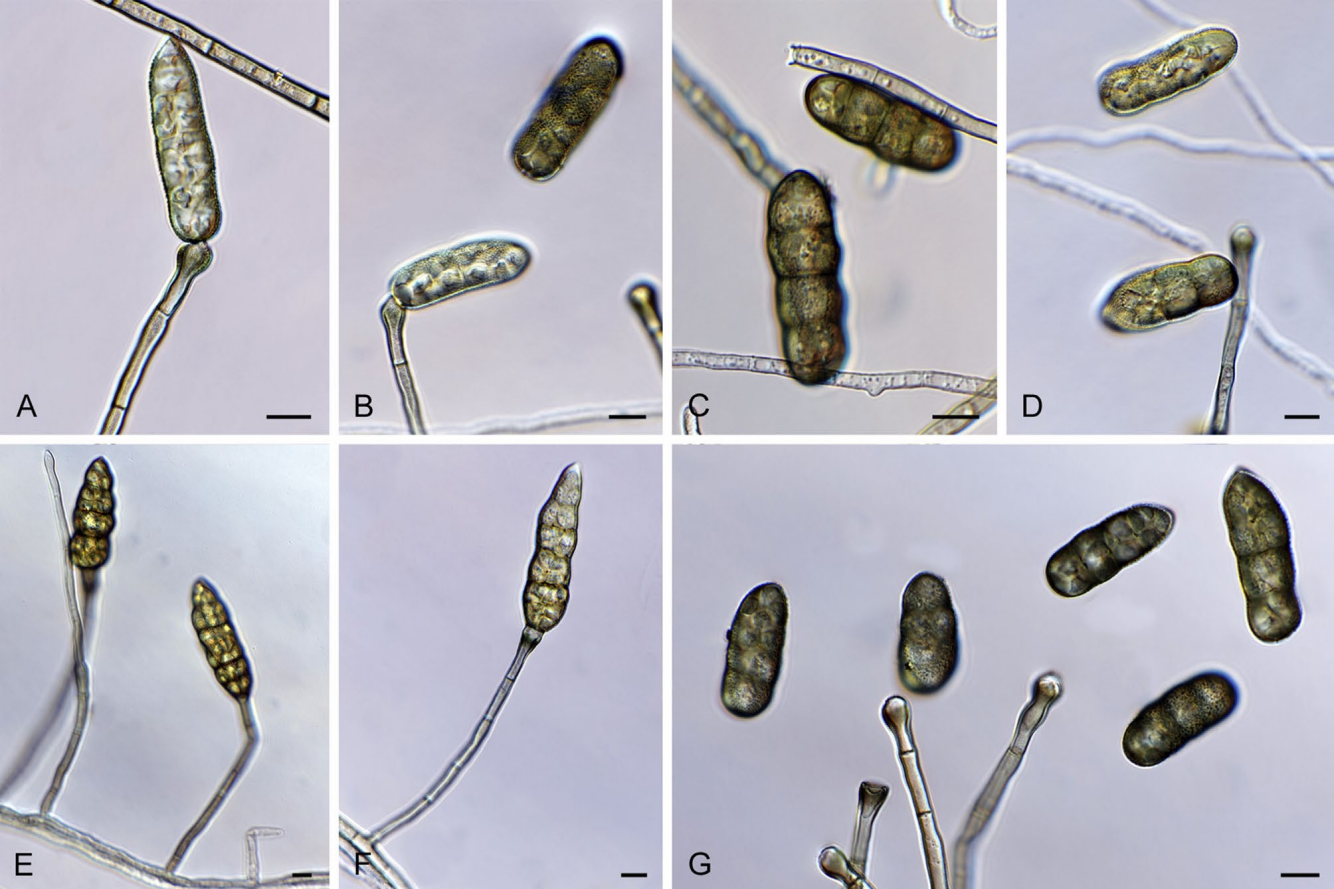
*Stemphylium lycopersici* conidiophores and conidia after 7 d on PCA. Reprinted with permission from Woudenberg & Crous, ‘Stemphylium revisited’, *Studies in Mycology*, vol. 87, no. 1, p.77–103, 2017. Copyright 2024 by the Westerdijk Institute of Fungal Biodiversity

This current case highlights rare and important issues in the field of infectious diseases; (1) the mixed infection caused by a rare fungus and a virus, and (2) the mNGS examination can rapidly and accurately identify pathogens and reveal changes in pathogen dominance. For the former issue, corneal scraping mNGS identified the fungal species *S. lycopersici*, contributing to the first reported case of keratitis caused by this pathogen. *S. lycopersici*, originally isolated from Solanum lycopersicum in Japan [18], belongs to the order Pleosporales (subclass Dothideomycetes), the family Pleosporaceae, and the genus Stemphylium, which is a significant plant pathogen [19]. *Stemphylium spp.* are important fungi characterized by dark filamentous spores and have a wide global distribution. *Stemphylium spp.* hold a crucial place in the classification of hemipteran fungi and can be distinguished into 28 species based on gene regions. Woudenberg et al. have meticulously detailed the morphology of *S. lycopersici* in their study (Fig. 4) [20]. We hypothesized that *S. lycopersici* may have been introduced into the immunosuppressed cornea through an external foreign body. Additionally, the patient’s cornea was damaged by stone chips, which could alter the immune barrier on the ocular surface. This injury may have facilitated the development of mixed polybiotic infectious keratitis in this case. We believe that using mNGS examination of corneal scrapes to identify fungal species could help identify this new pathogen of keratitis.

In general, it is difficult for ophthalmologists to quickly identify pathogens and detect changes in the prevalence of pathogens in mixed infections by standard examination and clinical review. In instances of refractory infectious keratitis where empirical treatment fails, several possibilities should be considered: (1) incorrect diagnosis of the pathogen and subsequent drug selection, (2) the presence of a mixed infection involving multiple microorganisms, (3) evolving microbial resistance, (4) systemic diseases that compromise ocular surface immunity, and (5) patient drug adherence was poor. We eliminated (4) and (5) as causes since the patient lacked a history of systemic diseases affecting the immunity of the ocular surface and demonstrated consistent adherence to their medication regimens. In the case of (1) and (2), the mNGS should be used to quickly identify all pathogens, enabling timely adjustments to the treatment plan. For scenario (3), the mNGS can discern the pathogenicity of microorganisms in infectious keratitis and pinpoint dominant shifts, thus facilitating prompt medication modifications. Furthermore, if feasible, microbial scraping should be performed for drug susceptibility testing. In this case, given the negative corneal scrape culture result, we used IVCM to diagnose a fungal infection and administered the appropriate empiric treatment. However, the inability to identify the specific type of fungus and diagnose mixed infections led to suboptimal treatment results. Thus, mNGS testing was conducted, which precisely identified the types of pathogenic microorganisms and diagnosed mixed infection. When presenting the results, the scientific method of mNGS minimizes the influence of contamination and eliminates the influence of the common ocular flora, significantly enhancing the reliability of the findings.

### Challenges in clinical and microbiological diagnosis

IK is an ophthalmic emergency that can cause significant corneal damage, leading to corneal opacification, and is one of the leading causes of blindness worldwide [1]. Early identification of the pathogen responsible for corneal infection allows rapid and optimal treatment, preventing irreversible corneal damage and significantly improving the chances of full recovery. However, clinical features are not specific to the type of microbial keratitis, making the early accurate diagnosis of IK challenging [21]. Consequently, both in vivo and in vitro tests are necessary to aid in the early diagnosis of IK. Corneal smears and cultures remain the gold standard for IK diagnosis; however, these methods lack sensitivity. Fungal cultures are particularly time-consuming, and some rare species require two weeks or more to confirm the presence or absence of pathogen growth [22]. Other widely used diagnostic modalities, such as direct microscopy, IVCM, and histopathological examination, also have significant drawbacks. These methods often result in a low positive rate for early diagnosis and may overlook mixed infections, preventing patients from receiving effective treatment promptly and leading to severe consequences [23]. The emergence of newer technologies highlights the inadequacies of older methods, which do not provide accurate and timely diagnoses necessary to guide treatment and preserve patients’ vision.

The patient had previously been treated in a local hospital before seeking care in our facility. Due to the inability to identify the pathogen, empirical treatment with levofloxacin eye drops tobramycin eye drops, and ganciclovir ophthalmic gel was administered. This approach masked clinical symptoms and delayed the accurate diagnosis and treatment of the disease. A corneal scraping culture yielded negative results and an IVCM examination suggested a fungal infection. Although many clinical features of fungal keratitis overlap, identifying the specific fungal species is crucial to guide the appropriate treatment. There are no distinct ocular signs to differentiate the rare fungal keratitis caused by *S. lycopersici* from other types of fungal keratitis. Empirical treatment with 5% natamycin eye drops was initiated, but the response was poor. Subsequent corneal scraping mNGS identified a mixed infection caused by *S. lycopersici* and *human alphaherpesvirus 1*.

### Challenges in clinical treatment

Broad-spectrum topical antibiotics are commonly used by clinicians to treat corneal infections of unknown etiology [24]. However, its extensive use alters the microbial spectrum and the respective antibiotic susceptibility patterns, potentially increasing the prevalence of drug-resistant bacteria that cause disease [25]. AMR has become a major public health threat in the 21 st century, driven by various factors, including diagnostic uncertainty leading to inappropriate antibiotic use [26]. In the current ‘post-antibiotic’ era, clinicians must exercise greater caution when choosing antibiotic therapy [27].

Ray et al., in their SCUT trial, discovered that the minimum inhibitory concentration (MIC) of bacteria isolated from patients with IK who had been pretreated with topical fluoroquinolones was 3.48 times higher than in those who had not received such treatment [28]. A subgroup analysis of the Mycotic Ulcer Treatment Trial I (MUTT I) also revealed that patients with IK treated with topical antifungal medications experienced a mean increase in MIC of 2.14 times per year, even after adjusting for pathogen factors [29]. Emerging technologies such as mNGS and artificial intelligence-assisted platforms improve the microbiological diagnosis of IK [30, 31], thus improving treatment and reducing the risk of AMR.

## Conclusions

To the best of our knowledge, this is the first documented case of fungal keratitis caused by S. lycopersici. It is also the first case of mixed infectious disease caused by a rare fungus with one virus. This case highlights the pivotal role of molecular diagnostics in the identification of human keratitis, especially in the increasing issue of drug resistance in pathogenic microorganisms. mNGS offers rapid and precise diagnostics, preventing the misidentification of uncommon pathogens and mixed infections, thus improving the clinical management of the patient’s vision. Additionally, prompt and accurate diagnostic results can inform clinical treatment strategies, mitigating the risk of increased drug-resistant organisms due to the misuse of antibiotics due to diagnostic ambiguity. Thus, in cases of complex corneal infections in which the pathogen is unclear, clinicians should consider performing mNGS on clinical samples as early as possible. This approach enables early identification of the causative microorganism and helps guide the appropriate treatment. However, it is important to note that traditional microbiological diagnosis remains essential to identify infectious keratitis. This case highlights the need for clinicians to use mNGS in refractory infection cases to achieve diagnostic precision and to tailor therapeutic approaches effectively.

## Data Availability

No datasets were generated or analysed during the current study.
